# Occupational Therapy Education in Saudi Arabia: Barriers and Solutions From a Cross-Sectional Survey Study

**DOI:** 10.7759/cureus.36139

**Published:** 2023-03-14

**Authors:** Muhammad O Al-Heizan, Saad A Alhammad, Mishal M Aldaihan, Khalid S Alwadeai

**Affiliations:** 1 Department of Rehabilitation Sciences, College of Applied Medical Sciences, King Saud University, Riyadh, SAU

**Keywords:** medical education, higher education, saudi arabia, health education, occupational therapy

## Abstract

Objective

Occupational therapy (OT) is an important healthcare profession in Saudi Arabia (SA). Yet, no studies have explored the status of OT education in SA. The current study aims to investigate the status of OT education in SA, as well as the barriers and solutions.

Methods

A cross-sectional-based survey was conducted. Institutions that offered OT programs were identified and an electronic survey was sent to OT program directors across SA that agreed to participate. The survey included items that focused on the number of students in programs, current faculty members, and their characteristics, as well as barriers and solutions to advancing the OT profession and education in SA.

Results

Out of 74 institutions, eight offered OT programs and all responded to the survey. Among all programs, one was inactive, seven (87.5%) were governmental institutions, and none were nationally accredited. There was a total of 538 currently enrolled students and 76 full-time faculty members. There are no current OT postgraduate programs. Staff shortages and ineffective communication between institutions (87.5%), financial barriers, and lack of knowledge/awareness of the OT profession (75%) were the most common barriers reported.

Conclusion

OT education is growing in SA but is still not well-represented throughout the different regions of the country. Initiatives to advance the profession are urgently needed by establishing new OT programs and departments as well as introducing OT to more diverse scopes of practice and in more clinical settings. Further research exploring OT education including curriculum content, teaching methods, and assessment strategies of OT programs. Addressing the barriers identified in the current study and methods to overcome them is needed.

## Introduction

Occupational therapists are client-centered healthcare professionals that focus on the promotion of health and well-being. Generally, occupational therapists help individuals across the lifespan to participate in daily occupations through the therapeutic use of everyday activities [1]. Occupational therapy (OT) as a healthcare profession first started in the US in the 1910s, treating world-war I veterans [2]. Since then, an expansion of the scope of OT practice has taken place.

In Saudi Arabia (SA), the Ministry of Health (MoH) was founded in the late 1950s, however, the first OT clinical service most likely started in Riyadh during the early to late 1980s. The first OT department in SA was started by the Medical Services of the Ministry of Defense (MoD), which is where the early Saudi OT workforce was trained. Consequently, various initiatives were introduced, including establishing new OT programs and sending students to study OT abroad to assure that qualified occupational therapists meet the increased demand for OT in SA. The first occupational therapy program within a university was established at King Saud University, and other universities quickly followed. In Saudi universities, educational programs require the approval of the university’s council to be established. The National Center for Academic Accreditation and Evaluation (NCAAA) accredits academic programs. OT graduates are then registered to practice by the Saudi Commission for Health Specialties (SCFHS).

An important milestone for the profession was the recognition of OT as a standard in clinical settings by the Central Board for Accreditation of Healthcare Institutions (CBAHI); this required healthcare institutions to establish more OT departments to receive national healthcare accreditation [3]. Further, the demand for more qualified OTs resulted in the SCFHS establishing a higher diploma for OT practitioners to gain advanced training in specified OT practice areas [4]. In fact, the demand for OT services in SA is increasing given the increasing national and regional prevalence rates of disability, which in turn leads to a healthcare and economic burden [5]. According to the most recent national survey, more than half a million Saudi citizens (1 out of every 30) reported the presence of a disability [5]. Further, there are increases in the rates of road traffic accidents with consequences of traumatic brain injuries, spinal injuries, fractures, amputation, and others [6,7]. There are also higher prevalence rates of strokes, cerebral palsy, and autism among the Saudi population [8-10]. Moreover, chronic health conditions have contributed to the increased disability rates in SA [11]. The MoH currently has a capacity of 235 rehabilitation beds and 705 long-term rehabilitation beds per 100,000 and plans to increase that capacity to 3190 beds and 3115 beds, respectively, by the year 2030 [12]. Therefore, the demand for OTs is high due to their vital role in addressing the high rates of disability in SA as well as the increase in rehabilitation and long-term rehabilitation demands currently and in the future.

Given the increasing demand for OTs and their important role in current healthcare practices, there is a need for a better understanding of the education of OTs in SA. This would inform future initiatives and policies to improve OT education and address the growing demand for OT services in SA. However, OT education in SA is yet to be explored to offer a better understanding of the current status and plans following the Saudi Vision 2030, which emphasizes the “alignment of educational outputs with labor market needs” [13]. Further understanding of the status and difficulties faced by OT programs is needed as well as the potential solutions. Therefore, the objective of the current study is to investigate and assess the status of OT education programs, teaching and faculty characteristics, and possible barriers and solutions.

## Materials and methods

The study utilized a cross-sectional design. Data used for the study were collected using a descriptive survey that was distributed between October and November of 2022. The electronic survey was developed to investigate the status of OT educational programs in SA. To assure the face and content validity of the survey, two members of the research group pretested and piloted the survey. We utilized the feedback to enhance the survey’s questions prior to data collection. The official website of the Ministry of Education (MoE) was used to search for institutions that had an OT department or program. In addition, the official website of the MoD was searched for any additional academic OT programs. In cases of institutes with multiple campuses, we considered each campus as a separate institution to investigate the geographic spread of current OT programs. After agreeing to participate, each program director of the identified institutions received a survey that focused on currently offered academic programs; faculty members employed by the program (full-time and part-time); faculty members' characteristics, including their level of education, work allocation, scholarship status, and gender. For the current study, anyone that participated in the educational process, including lectures, laboratories, or clinical training, was considered a faculty member. In addition, the survey included questions that focused on the status of accreditation, techniques used in teaching, and laboratories used for teaching clinical competencies. Finally, questions that focused on the barriers and potential solutions to advancing the OT profession in SA were included.

Data collection and analysis

Outlook Forms (Microsoft Corporation, Redmond, WA) was used to collect data, and Microsoft Excel (Office 365; Microsoft Corporation) software was used to export and compile the data. Given the nature of the survey, we used frequencies, percentages, and measures of central tendency to describe survey results. Due to the cumulative nature of the data collected, no statistical analysis was performed.

## Results

Of the 74 universities and colleges that were searched through the websites of the MoE and the MoD, we identified only eight OT programs, and one was within a private institution. Table 1 gives an overview of the institutions. All program directors were contacted, and all completed the survey.

**Table 1:** Occupational Therapy Public and Private Institutions Across Saudi Arabia ^π ^Private Institution ^a ^Inactive (No Students are currently enrolled)

#	University	City, Region	Students		Program Launch Date
			Male	Female	
1	Batterjee Medical College^π^	Jeddah, Makkah	√	√	2019
2	King Abdulaziz University	Jeddah, Makkah	√	√	2019
3	King Saud Bin Abdulaziz University for Health Sciences	Alhafouf, Eastern region	√	-	2018
4	King Saud Bin Abdulaziz University for Health Sciences	Jeddah, Makkah	√	√	2017
5	King Saud Bin Abdulaziz University for Health Sciences	Riyadh, Riyadh	√	√	2012
6	King Saud University	Riyadh, Riyadh	√	√	2010
7	Northern Border University^a^	Arar, Northern Border region	-	-	2022
8	Princess Nourah Bint Abdulrahman University	Riyadh, Riyadh	-	√	2013

The overall characteristics of the institutions are highlighted in Table 2. Out of 13 regions in SA, nine regions (69.23%) do not have an institution currently offering an OT educational program.

**Table 2:** Characteristics of the institutions ^π ^13 provinces were combined based on their geographic locations into five regions. Central: Riyadh and Qassim. Eastern: Eastern region only. Western: Makkah and Madinah. Southern: Aseer, Albaha, Jizan, and Najran. Northern: Hail, Aljouf, Tabuk, and Northern Border region ^a ^Median Full-time faculty only, one institution did not report their faculty-student ratio

Characteristics	N (%), N (M/F), Mean ± SD
Geographic regions^π^	
Central	3 (37.5%)
Eastern	1 (12.5%)
Western	3 (37.5%)
Southern	0 (0.00%)
Northern	1 (12.5%%)
Active programs	7 (87.5%)
Sector	
Governmental	7 (87.5%)
Private	1 (12.5%)
Student	
Graduated over the past 5 years	606 (252 /354)
Currently enrolled	538 (187/351)
Expected to graduate in 5 years	1086 (433/653)
Faculty member characteristics	
Full-time	76 (92.68%)
Part-time	6 (7.32%)
BSc holders	22 (32.83%)
MSc holders	34 (50.75%)
PhD holders	11 (16.42%)
MSc scholarship	16 (41.02%)
PhD scholarship	23 (58.98%)
Faculty member-student ratio^a^	1:13
Accreditation and recognition	
NCAAA obtained	0 (0%)
NCAAA in-process	4 (50%)
International recognition (WFOT)	5 (62.5%)
No accreditations were pursed	2 (25%)
Education degrees and styles	
Diploma degree offered	0 (0.00%)
BSc degree offered	8 (100%)
MSc or PhD degree offered	0 (0.00 %)
Clinical hours apart from internship	20.62 ±10.15
Research course as part of the curriculum	8 (100%)
Implementation of problem-based learning	7 (87.5%)
Implementation of clinical simulation in teaching	7 (87.5%)

No institutions offered a diploma degree. All eight institutions offered an undergraduate (BSc) degree, and no institutions offered a postgraduate degree (either Master of Science (MSc) or Doctor of Philosophy (PhD)). The eight institutions reported that they had 606 students that have graduated over the past five years. During the time of the survey, 538 students were enrolled in OT programs. Program directors indicated that they expect 1086 students to graduate from their programs in the upcoming five years. The current study revealed that more female students graduated in the past five years (58.41%), are enrolled currently (65.24%), and are expected to graduate in the upcoming five years (60.12%) as compared to their male counterparts.

There were 76 full-time faculty members with various degree levels and only six faculty members were part-time. Thirty-nine faculty members are on a current scholarship to pursue a postgraduate degree, 16 (41.02%) were currently pursuing an MSc degree, and 23 (58.98%) were pursuing a PhD degree. The current level of education of faculty members revealed that 22 (32.83%) held a BSc degree, 34 (50.75%) held an MSc degree, and 11 (16.42%) held a PhD degree.

None of the eight institutions reported being accredited by the NCAAA. Four (50%) institutions reported that the NCAAA accreditation was in process, however, two (25%) institutions reported that no accreditation was being pursued at the time of completing the survey. At an international level, five (62.5%) institutions reported being recognized by the World Federation of Occupational Therapists (WFOT), although none were accredited by NCAAA.

**Figure 1:**
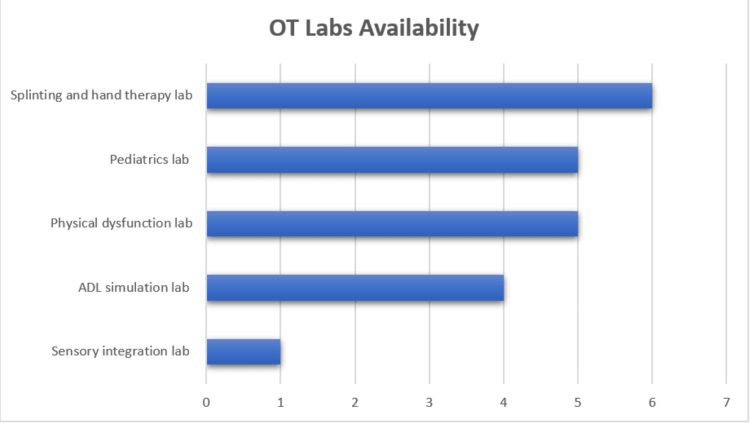
Occupational therapy laboratories that institutions are utilizing in teaching and training

All eight institutions state that their curriculum has a research course. Further, problem-based learning, as well as clinical simulations in teaching, is implemented in seven (87.5%) institutions. Only one (15.38%) institution did not have any laboratories at the time of the survey, which was an inactive program; however, they reported that laboratories are currently being developed. The program directors reported the following laboratories are being used in the teaching of their students: six (75%) splinting and hand therapy laboratories, five (62.5%) physical dysfunction and pediatrics laboratories, four (50%) activities of daily living (ADL) simulation laboratories, and one (12.5%) sensory integration laboratory. Figure 1 shows the different laboratories and the number of institutions that utilize them.

The overall faculty-student ratio had a range from 1:6-1:17, with a Median ratio of 1:13. Program directors reported an expected growth of over 50% in enrollment in the upcoming five years. However, current faculty members on scholarship are expected to join the OT programs, resulting in an increase in the current faculty workforce and a reduction in the overall faculty-student ratio.

The average number of clinical hours apart from the internship was 20.62 ± 10.15 with a range of 10-36. Six institutions (75%) reported that they faced difficulty finding clinical sites to provide clinical placements for students. Moreover, all eight institutions reported that they faced difficulty finding clinical sites to provide clinical rotations for student interns. Program directors reported possible solutions for easing these difficulties, including communicating with stakeholders in different private and governmental sectors and scopes of practice to increase and expand OT departments and positions, equal distributions of internships in all hospitals, and decreasing the length of internships.

**Figure 2:**
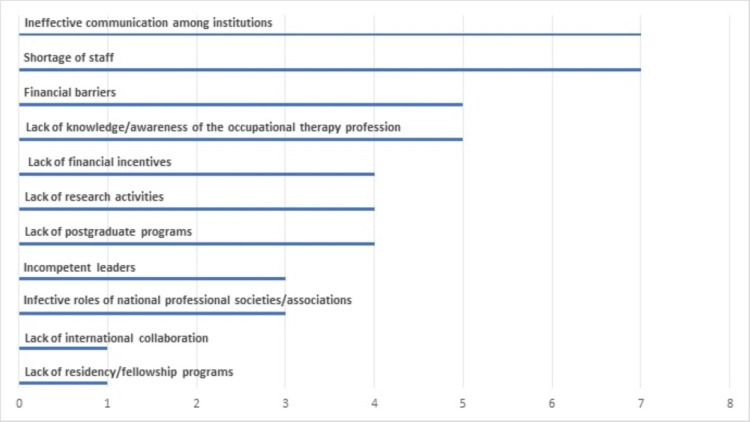
Barriers or limitations of advancing the occupational therapy profession

The program directors reported a variety of barriers that hinders the development of the OT profession in the country, which included ineffective communication between institutions and shortage of staff (n=7) 87.5%, financial barriers, and lack of knowledge/awareness of the OT profession (n=6; 75%), lack of research activity, financial incentives, and postgraduate programs (n=4; 62.5%). Figure 2 shows the barriers to developing the OT profession and the number of responses.

**Figure 3:**
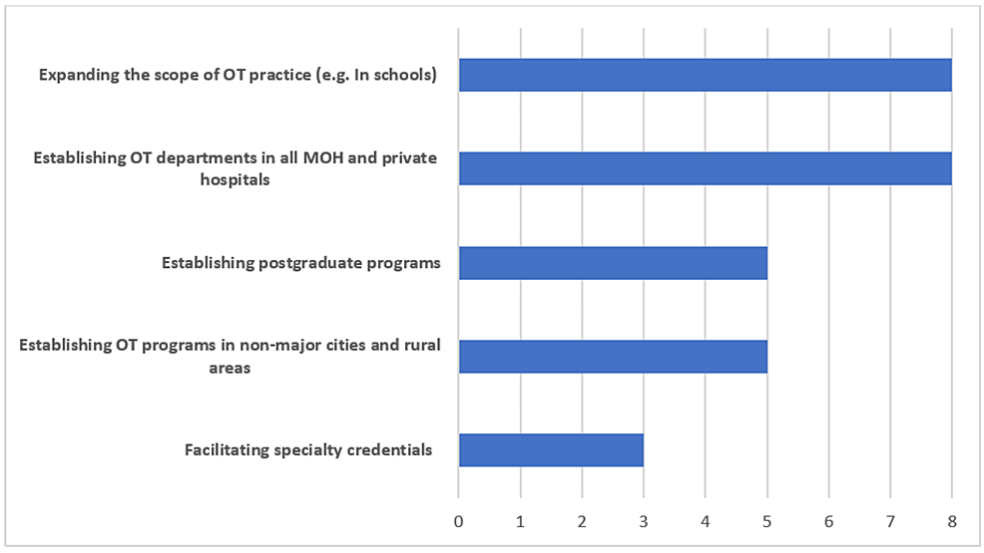
Proposed solutions for expanding the occupational therapy profession

In addition, program directors reported the following as proposed solutions for expanding the OT profession: the establishment of OT programs in both governmental and private sectors (n=8) 100% and the expansion of the current scope of OT practice (n=8) 100%. In addition, some of the possible reported solutions included facilitating inter-professional education, increasing funding to new OT departments, increasing the number of OT scholarships, establishing scope of practice and professional guidelines. Figure 3 shows the proposed solutions to the barriers.

## Discussion

The current study is, to our knowledge, the first to explore the status of OT education in SA. OT education in SA is developing but is not well-distributed throughout the country. Most institutions are governmental, and all have not been nationally accredited by the NCAAA. During the time the study was conducted, the highest academic degree offered was an undergraduate degree. Compared to male students, female students are greater in number. Staff shortages, ineffective communication between institutions, financial barriers, and lack of knowledge/awareness of the OT profession were reported as barriers to developing OT in SA.

Out of 13 regions in SA, the Central region (Qassim), Northern regions (Hail, Aljouf, and Tabuk), Western region (Madinah), and Southern regions (Aseer, Albaha, Jizan, and Najran) do not have a current institution that offers an OT degree. Further, the Eastern region, a large region in SA, only had one institution offering an OT degree. OT programs not existing in those regions would most likely result in a smaller number of settings that provide OT services. Thus, it is essential to establish new OT programs within these regions, which might have several benefits, including covering the clinical demands and generating new opportunities for local OTs to provide high-quality OT services.

While there is high demand for OTs in clinical settings, there is also an increasing demand for OTs with postgraduate degrees in research, management, and academics. This is important to fulfill the leadership and educational demands of the expanding profession. Undergraduate degrees might not be sufficient in the development of leadership and advanced research skills as postgraduate degrees. Not to mention that postgraduate degrees may also result in the advancement of other important skills such as publishing, presenting, and teaching [14]. Moreover, the Accreditation Council for Occupational Therapy Education (ACOTE®) standards include knowledge of research methodology and the ability to apply evidence-based practice and review and critique scientific literature [15]. Although all eight current OT programs are reported to have curriculums with a research course, there is a need for better and more advanced research skills that would enable the enforcement of such standards in OT programs in SA. The current findings indicate that no academic postgraduate degrees in OT exist in SA. Nevertheless, efforts to fill this gap have been made through the establishment of a professional graduate OT diploma program by the SCFHS [4]. The program requires clinical rotations for two years in a variety of OT practice areas such as neurology, physical dysfunction, burns, and pediatrics. OTs are then credentialed as senior specialists after the program is completed.

Although the present study may indicate a low overall faculty-student ratio (1:13) with ratios ranging from 1:6 to 1:17, this ratio was higher than the ideal faculty-student ratio recently reported (1:10) [16]. Further, medical sciences education may require an even lower faculty-student ratio (1:7) [17]. In addition, most faculty were BSc or MSc degree holders, with a limited number of faculty members with a PhD, which may limit the scope and depth of education in most OT programs. A reduction in the current faculty-student ratio is recommended, particularly in health sciences education. This is particularly important given the expected increase in students’ enrollment in existing programs. Research suggests that reducing the faculty-student ratio is associated with improved performance in clinical exams [18]. This is also aligned with the programs’ director’s indication that faculty shortages as a barrier to the advancement of OT in SA.

Although four OT programs reported that they have international recognition from the WFOT, all OT programs lacked national accreditation from the NCAAA. Accreditation is considered a vital part of healthcare educational programs [19]. Lack of accreditation may impact the competency of graduates and result in variations in graduate skills. However, discrepancies in educational styles may also be a factor that influences graduate competencies. Therefore, standards of education should be established and applied by the educational programs, the NCAAA, and the SCFHS to continuously assure the quality of OT educational outcomes. The current study did not evaluate the alignment of current OT programs with WFOT educational standards; however, this should be addressed in future studies. The discrepancies in laboratories utilizations may have also been a result of the lack of accreditation, which is important in the development of students’ knowledge and skills, particularly clinical skills. Some programs lacked crucial laboratories such as ADL simulation laboratories. Furthermore, some institutions had a limited number of laboratories, which may result in decreased confidence or clinical competence, which may, in fact, result in significant barriers for students during clinical training.

Clinical placements were also an area of concern for most OT programs, with 75% of programs reporting that they face difficulty finding clinical sites to provide clinical placements for students and all programs reporting that they face difficulty finding clinical sites to provide clinical rotations for student interns. Recently, difficulties with the clinical placement of OT students have been reported [20]. This has been attributed to the restructuring of healthcare, shortages in staff, and increased number of enrollments in health profession programs [21]. Program directors reported the need for the expansion of current and opening of new OT departments is essential through advocacy and communication with stakeholders, including in non-major cities and rural areas. In addition, there is a dire need for expanding the current scope of OT practice to include new areas, such as schools, community centers, nursing home facilities, and home health, which has also been suggested as an important solution for the advancement of the OT profession by OT program directors. Other suggestions include decreasing the current length of internship (one year) and moving to other methods for clinical placement such as simulation [22]. Most OT programs reported implementing problem-based learning as well as clinical simulation as part of their programs, both approaches have been suggested to improve clinical competence in medical sciences students [23,24]. The average number of clinical contact hours apart from the internship was 20.62 ± 10.15 with a range of 10-36. To our knowledge, there are no reported standards for clinical placement within OT educational programs, however, there were reported discrepancies among programs. This warrants the need for better educational standards for OT education to assure consistency and quality, which could be achieved through professional and/or academic societies.

To further describe the characteristics of the current OT programs in SA, program directors were asked questions regarding the barriers and suggested solutions to their current role as OT program directors. Shortages of staff and ineffective communication between institutions followed by financial barriers and lack of knowledge/awareness of the OT profession were the most frequently identified barriers. Staff shortages are a common barrier that has been reported before in the literature, even in countries where OT is an established profession [25,26]. The reported numbers of faculty members on either an MSc or PhD scholarship should help further increase the faculty workforce shortage. However, a noteworthy finding of the current study was the identification of issues in communication among institutions as a major barrier to advancing the OT profession. Collaboration and communication among institutions are important aspects of development, innovation, and attaining academic accreditation [27,28]. More collaboration among OT programs may benefit the advancement of the profession in the future.

Currently, there are no OT-specific postgraduate degrees in SA, and this finding has significant national implications for developing the OT profession by establishing postgraduate OT programs within different specialty areas. This would support the enhancement of OT research and, eventually, patient care by providing high-quality education and research at universities across SA. Further, promoting and funding students’ research and innovation activities is required to increase research engagement. This is crucial as research indicates that improved research activity was linked to better patient experiences and quality of care [29].

The establishment of OT programs in regions with a limited number of OTs is highly needed, particularly in the Eastern, Northern, and Southern regions. This is aligned with the strategic objectives of Saudi Vision 2030, which emphasizes the importance of achieving equality of access to education, particularly in less developed areas of the country, which would improve healthcare services, and achieve a prosperous economy and society. This was also highlighted as a possible solution for expanding the OT profession by OT program directors in this study. Finally, since OT is a necessary aspect of healthcare practice in SA, it should be enabled by the promotion and implementation of a variety of OT areas of specific clinical practice to enhance the care of patients and service delivery.

Limitations

This study is the first step toward gaining in-depth knowledge of the status of OT education in SA. However, there are noteworthy limitations that were encountered: information regarding current students studying OT abroad is unknown, information regarding the educational background of current OT faculty members, input from the healthcare industry regarding the education of OTs is lacking, and we did not evaluate the differences between OT curriculums or teaching and assessment methods among programs. We also did not collect information regarding any future upgrades to current OT programs to get a sense of future trends. Furthermore, we did not have access to data on the current work location of previous OT program graduates, which may have limited our ability to assess geographical relocation.

## Conclusions

OT education in SA has seen significant improvements; still, many areas have opportunities for further progress. The Eastern, Northern, and Southern regions of SA are underrepresented, and the development of OT programs may help healthcare providers in those regions deliver OT services. Further, the introduction of OT in other scopes of practice such as schools, community centers, nursing home facilities, and home health is indicated. Given that all institutions did not have national accreditation, the quality of OT programs is a significant area of concern. Improving communication between institutions and stakeholders is important, such as health services, to safeguard better clinical training and increase the need and quality of graduates. There is a need for well-established postgraduate programs to help advance the OT profession in SA. The new professional OT postgraduate diploma program developed by the SCFHS offers a great start that should be built upon for the future. Further research is needed to provide a more detailed and comprehensive understanding of current programs and the barriers identified in the current study to help further advance OT education in SA.
